# Love Lost in Translation: Avoidant Individuals Inaccurately Perceive Their Partners’ Positive Emotions During Love Conversations

**DOI:** 10.1177/01461672241258391

**Published:** 2024-07-27

**Authors:** Stéphanie E. M. Gauvin, Jessica A. Maxwell, Emily A. Impett, Geoff MacDonald

**Affiliations:** 1 Acadia University, Wolfville, Nova Scotia, Canada; 2 McMaster University, Hamilton, Ontario, Canada; 3 University of Toronto, Ontario, Canada

**Keywords:** adult attachment, communication, intimacy, multilevel modeling, romantic relationships

## Abstract

Empathic accuracy—the ability to decipher others’ thoughts and feelings—promotes relationship satisfaction. Those high in attachment avoidance tend to be less empathically accurate; however, past research has been limited to relatively negative or neutral contexts. We extend work on attachment and empathic accuracy to the positive context of love. To do so, we combined data from three dyadic studies (*N* = 303 dyads) in which couple members shared a time of love and rated each other’s positive emotions. Using the Truth and Bias Model of Judgment, we found that individuals higher (vs. lower) in attachment avoidance were less accurate in inferring their partners’ positive emotions during the conversation, but did not systematically over- or under-perceive their partners’ positive emotions. Our results suggest that avoidant individuals may be less sensitive to positive cues in their relationships, potentially reducing relational intimacy.

“It’s so easy, to think about love, to talk about love, to wish for love, but it’s not always easy, to recognize love, even when we hold it . . . in our hands.” (Jaka, n.d.)

Affectionate communication—including sharing one’s feelings of love, closeness, and support for a partner—plays a pivotal role in the development and maintenance of romantic relationships (e.g., Floyd, 2002; see review by Le et al., 2010). But what if these messages of love become lost in translation? Beyond talking the talk, *showing* love to one’s partner, such as smiling, leaning in, touching, and increasing gaze, is a powerful signal of intimacy (Andersen et al., 2006). However, individuals must decode these signals to accurately infer love from the content and emotional tone of what their partner is communicating. Accurately inferring love (i.e., empathic accuracy) may be particularly important during the early stages of relationships by promoting intimacy, effective communication, and cooperation during initial relational challenges (see Sened et al., 2017 for a review). Given the important role that satisfying and fulfilling romantic relationships play in promoting physical, emotional, and psychological well-being (e.g., Diener & Seligman, 2002; Holt-Lunstad et al., 2010), it is important to understand how individuals communicate their love and affection in relationships, and identify who may struggle with decoding these signals of love. We suggest that individuals higher in attachment avoidance—those who tend to avoid emotional closeness with romantic partners (see review by Mikulincer & Shaver, 2016)—may struggle to decode their partners’ love.

## Attachment Avoidance

Attachment theory suggests that security or insecurity with the pursuit of interpersonal closeness develops in response to an individual’s success—or lack thereof—in seeking and receiving comfort from attachment figures (Bowlby, 1969). Adults demonstrate reliable individual differences in attachment security along dimensions of attachment anxiety and avoidance (Fraley, Waller, & Brennan, 2000), where individuals high in either form of attachment insecurity hold maladaptive interpersonal schemas (Beck, 1987)—or *negative working models.* For individuals higher in attachment anxiety, these negative working models are associated with low self-efficacy for emotionally coping without close others, but a hesitation to approach closeness due to fears of rejection (Shaver & Mikulincer, 2002). For individuals higher in attachment avoidance (i.e., avoidant individuals), these negative working models are associated with expectations of unrewarding or even negative relational outcomes (e.g., seeing others as less caring and responsive). They perceive expressions of closeness as somewhere between pointless and dangerous, believing that seeking proximity to an attachment figure will not reduce emotional distress and instead may result in punishment such as loss of autonomy. As such, avoidant individuals learn to cope with distress by deactivating their attachment systems and disregarding or suppressing negative feelings, which in theory should reduce motivation to turn to an attachment figure (e.g., Edelstein & Gillath, 2008; Fraley & Shaver, 1997). Many researchers theorize that avoidant individuals employ various defensive strategies to reduce attachment system activation (e.g., Main & Weston, 1982; Overall & Simpson, 2015; Shaver & Mikulincer, 2002).

Behaviors that avoidant individuals exhibit that may function to reduce activation of their systems include tuning out attachment-relevant information (e.g., Dewitte et al., 2007; Edelstein & Gillath, 2008; Rholes et al., 2007, see review by Shaver & Mikulincer, 2002), and encoding less information about attachment-relevant experiences into memory (Fraley & Brumbaugh, 2007; Fraley, Garner, & Shaver, 2000). Although replication is needed, these findings suggest that avoidant individuals are less sensitive to attachment-related information, which might serve to minimize attachment system activation.

Because the attachment system is theorized to become active in response to emotional experiences that may otherwise promote closeness (Shaver & Mikulincer, 2002), avoidant individuals may default to disengaging from emotional stimuli (e.g., Dewitte, 2011; Dewitte & De Houwer, 2008; Fraley, Garner, & Shaver, 2000; cf., Sanscartier et al., 2020). Avoidant individuals may be particularly insensitive to positive emotions (Mikulincer & Sheffi, 2000; Park et al., 2023; Vrtička et al., 2008). In fact, attachment avoidance is related to lower expectations (Spielmann et al., 2013) and experiences (Gere et al., 2013) of intimacy in romantic relationships. Likewise, when asked to recall their earlier reactions to daily events, those higher in attachment avoidance underestimate the positive emotions they felt, specifically for interpersonal events (Gentzler & Kerns, 2006).

Of interest, such difficulties with attending to and decoding positive emotions may be most pronounced in intimate relational contexts (Park et al., 2023). Indeed, avoidant individuals in relationships show poorer decoding for a stranger’s positive emotional expressions than those who are single, suggesting avoidant defenses may be particularly triggered in close relationships (Kafetsios et al., 2014). Collectively, the lack of engagement with emotional stimuli may have consequences when interacting with a romantic partner; in particular, avoidant individuals may have difficulty accurately inferring their partners’ emotions (i.e., empathic accuracy).

## Empathic Accuracy

The ability to accurately infer the content of another person’s thoughts and feelings —*empathic accuracy* (Ickes, 1993)—is important to interacting successfully with others in a variety of life domains as it prevents misunderstandings (Ickes & Simpson, 1997, 2001) and promotes satisfaction in interpersonal interactions (Lun et al., 2008). In romantic relationships, empathic accuracy is associated with effective couple communication (Noller & Ruzzene, 1991), enhanced couple well-being, cooperation during conflict (Kilpatrick et al., 2002), skillful support provision (Verhofstadt et al., 2008), and relationship satisfaction (see review by Sened et al., 2017). Individuals who are less adept at interpreting their romantic partners’ feelings are not afforded these relationship benefits, and hence face poorer relationship functioning.

Although empathic accuracy is primarily advantageous to one’s romantic relationships, there are instances in which correctly inferring one’s partner’s thoughts is detrimental. For instance, in situations in which a partner’s thoughts are relationship-threatening (e.g., when a partner is viewing an attractive potential mate), greater accuracy reduces intimacy and increases instability between partners (Simpson et al., 2003). According to Ickes and Simpson’s Empathic Accuracy Model (Ickes & Simpson, 1997, 2001), every couple has domains in their relationship in which understanding a partner’s feelings is particularly painful (referred to as “danger zones”). These danger zones might vary by the individual (e.g., between men and women, across attachment styles, Ickes & Simpson, 1997, 2001). For example, some individuals might find relationship-threatening contexts, like having a romantic partner talk about an ex or an attractive person, to be a “danger zone” (e.g., Hussain et al., 2021). The Empathic Accuracy Model (Simpson et al., 1995, 1999; see Ickes & Simpson, 2001, pp. 229–249 for a review) posits that individuals first try to avoid or exit situations where they might encounter a danger zone; however, if they cannot avoid the threatening situation then their next defense is motivated empathic inaccuracy.

## Attachment and Empathic Accuracy

An individual’s attachment orientation likely influences what contexts they construe as danger zones, and how they strategically manage their empathic accuracy in these danger zones. Secure individuals (scoring low on the avoidance and/or anxiety dimension) manage their empathic accuracy in romantic relationships in an arguably adaptive fashion according to the threat level of the situation (Ickes, 2003): They are more accurate in low threat situations, but less accurate during high threat situations (e.g., Dugosh, 1998; Simpson et al., 1999), such as discussing a high severity intimacy issue (Simpson et al., 2011). In contrast, those high in attachment avoidance seem to be less empathically accurate regardless of threat level; for example, across discussions of both severe and minor intimacy and jealousy-related problems with their partner (Simpson et al., 2011) and even when interacting with strangers (Izhaki-Costi & Schul, 2011; Kafetsios et al., 2014).

However, it is unclear in these studies *how* more avoidant individuals were less accurate. As noted by Overall et al. (2015), one reason avoidant individuals may be less accurate is that they inhibit attention to their partner, meaning they are poor at tracking their partners’ thoughts and feelings (Bowlby, 1980). Lower accuracy may also stem from negative schema-driven information processing, meaning avoidant individuals overestimate how negative—or underestimate how positive—their partner is feeling (Bowlby, 1973), referred to as a negative mean-level/directional bias. That is, avoidant individuals may be poor at inferring their partner’s feelings because (a) they do not notice fluctuations in these emotions, and/or (b) because they see everything through a pessimistic lens. Advances in statistical and theoretical modeling, namely the introduction of the Truth and Bias Model of Judgment (T. V. West & Kenny, 2011) provided Overall et al. (2015) with the opportunity to tease apart these two processes—tracking accuracy versus mean-level/directional bias (see also Fletcher & Kerr, 2010). They assessed partners’ true negative emotions at several points during a conflict discussion, and at the end of each day for three weeks, and compared these to avoidant individuals’ perceptions of their partner’s emotions. They found that individuals higher in avoidance noticed shifts in their partners’ negative emotions (i.e., they accurately tracked their partners’ negative emotions); however, they consistently overestimated the intensity of their partners’ negative emotions. Thus, these results suggest that avoidant individuals’ lower accuracy is not due to inhibited attention (because they accurately tracked their partner’s negative emotions), but rather, avoidant individuals process information through negative schemas. This study highlights the importance of disentangling poor tracking from the tendency to overestimate or underestimate to identify whether avoidant individuals have a deficit in the tendency to attend to a partner’s emotions, whether they pick up the information correctly but filter it through a pessimistic lens (see also Sadikaj et al., 2018), or whether both processes relate to reduced accuracy. Like Overall et al. (2015), in this study, we harness the Truth and Bias Model of Judgment to differentiate these nuanced processes.

### Truth and Bias Model of Judgment

We use the Truth and Bias Model of Judgment (T. V. West & Kenny, 2011) to operationalize empathic accuracy, which affords us several advantages over past work on empathic accuracy and attachment (e.g., Simpson et al., 2011). Notably, this model will allow us to see whether attachment avoidance predicts the extent to which participants correctly infer their partners’ positive emotions (i.e., accuracy, or “truth,” denoting how closely associated one’s perceptions are with a partner’s true scores) and/or whether they underestimate their partners’ emotions (i.e., a negative directional bias). These two estimates will allow us to disentangle attentional biases (which would result in low tracking accuracy) from negative schemas (which would result in a directional bias; Overall et al., 2015; Sadikaj et al., 2018). Importantly, our chosen framework accounts for the role of assuming similarity (i.e., projecting one’s own emotions when making judgments of a partner’s emotions; e.g., Lemay et al., 2007), thus ensuring that the effects of accuracy and directional bias are independent from the participants’ own emotional state. By simultaneously estimating directional bias, accuracy, and assumed similarity in the same model, we are assessing empathic accuracy in a way that we can disentangle between avoidants’ poor accuracy versus pessimism, and contextualize our findings within broader trends in the field (LaBuda & Gere, 2023).

### Avoidance and Empathic Accuracy in the Context of Positive Emotions

To date, the majority of research examining attachment and empathic accuracy in couple interactions has centered on negative relational contexts such as inferring negative emotions during conflict or threat (cf., Tucker & Anders, 1999). Yet, only examining attachment and empathic accuracy in threat situations is a critical gap since for avoidant individuals, signs of intimacy may be their true “danger zone.” Indeed, expressions of a partner’s love meant to foster intimacy when filtered through avoidant individuals’ negative schemas may instead foster feelings of discomfort and deactivate their attachment systems (Mikulincer & Shaver, 2007).

Consistent with this interpretation of intimacy as a “danger zone,” avoidant individuals tend to perceive fewer rewards in their relationship, possibly to guard against potential feelings of distress (Gere et al., 2013; MacDonald et al., 2013; Spielmann et al., 2013). In fact, avoidant individuals are generally less receptive to their partners’ messages of love (Schrage et al., 2020). Likewise, avoidant women experience fewer positive emotions when both sharing and hearing about positive daily events with their partners (Hicks & Diamond, 2008). Failing to capitalize on such relationship rewards is unfortunate, as there is a growing body of research that positive relationship factors promote positive outcomes for avoidant individuals (Park et al., 2019; Stanton et al., 2017).

## Current Study

Because intimacy is likely to threaten avoidant individuals’ desire for autonomy, but paradoxically is a chance for avoidant individuals to reap relationship rewards, it is important to understand how avoidant individuals pick up on their partners’ intimacy cues. Thus, the current research examines attachment and empathic accuracy for positive emotions during conversations in which both couple members share their love for each other.

### Overview of Study Hypotheses

Given evidence that avoidant individuals have difficulty both sharing and receiving positive events (e.g., Hicks & Diamond, 2008; Shallcross et al., 2011), we did not have clear a priori expectations for differences in effects between conversations (when the person was sharing or listening to their partner share love). As such, our predictions below pertain to both conversations.

#### Hypothesis 1: Directional Bias

As avoidant individuals have a negative working model/schema of others (and consistent with Overall et al., 2015; Sadikaj et al., 2018), we expect that individuals higher in attachment avoidance (+1*SD* above the mean) would underestimate their partners’ positive emotions during conversations about love. Statistically, this would be reflected in a negative main effect of attachment avoidance, followed by a negative intercept at high levels of avoidance (+1*SD* above the mean).

#### Hypothesis 2: Accuracy

The reviewed evidence examining empathic accuracy and attachment avoidance suggests that higher avoidance is associated with lower accuracy (i.e., a nonsignificant or weaker association between their partners’ true emotions and their perceptions of their partners’ emotions, reflected in a smaller Truth coefficient^1^), consistent with avoidant individuals’ tendency to be insensitive to attachment-related and emotional experiences (see Shaver & Mikulincer, 2002). During conversations about love, people high in avoidance may not attend to their partners’ emotions (e.g., Dewitte, 2011), or may attend to but then dismiss these emotions (e.g., Fraley & Brumbaugh, 2007), and hence fail to correctly perceive their partners’ positive cues of love. Thus, we hypothesize that individuals high in attachment avoidance (+1*SD* in simple slopes analyses) will show lower empathic accuracy in inferring their romantic partner’s positive emotions relative to those low in avoidance (−1*SD*).

#### Exploratory: Assumed Similarity

We have no strong a priori predictions regarding assumed similarity (also referred to as projection). On one hand, to facilitate emotional distance and avoid interdependence it is possible that avoidant individuals will assume their partner feels less similar to themselves than those who are lower in avoidance (e.g., Mikulincer et al., 1998). In other words, those high in avoidance may show weaker associations between their perceptions of their partners’ emotions and their own reported emotions, than those low in avoidance. Yet, on the other hand, it is possible that to best serve avoidant individuals’ goals of maintaining emotional distance, they would assume their partner felt similarly low levels of love to themselves during the conversation, which would closely resemble their own low feelings of positivity/intimacy in the relationship (e.g., Pietromonaco & Barrett, 1997). Such a pattern would be reflected in a stronger association between perceptions of a partner’s emotions and own emotions for those higher in avoidance. However, past research on perceiving negative emotions has found no effects of avoidance on assumed similarity (Overall et al., 2015; Sadikaj et al., 2018); thus, a null association is also possible.

## Method

Supplemental materials including analysis code, and data with a codebook are available on https://osf.io/ucbmr/?view_only=d3792deb1bf54098bd2e59ce81258f42.

### Participants

We recruited 303 couples across three sample sources (see also Schrage et al., 2020). We recruited Sample 1 (*N* = 124 couples) and Sample 2 (*N* = 100 couples) from the Greater Toronto Area through advertisements on the University of Toronto campus (undergraduate psychology courses, campus flyers), social media posts (Facebook groups), and classified ads (Kijiji.com and Craigslist.org). We compensated participants in Samples 1 and 2 with either one course credit or 20 CAD. We recruited Sample 3 (*N* = 79 couples) from the San Francisco Bay area (USA) through community flyers and classified ads (Craigslist.org), and compensated participants with 20 USD. No studies in this manuscript were preregistered, however, we report all manipulations,^2^ analyzed measures, and exclusions in these studies. We determined sample size based on broader study goals, resource constraints, and sample sizes in similar research at the time of data collection (e.g., Simpson et al., 2011). Although we did not calculate statistical power a priori, our sample of 303 dyads enables us to detect small actor and partner effects of *r* = .15 with 80% power^3^ (Ackerman & Kenny, 2016). Participant demographic and relationship characteristics are in Table 1.

**Table 1.** Sample Demographics and Descriptive Statistics.

Individual-level descriptive statistics	Sample 1 (*N* = 248)	Sample 2 (*N* = 200)	Sample 3 (*N* = 158)
Age (*M*(*SD*))	21.70 (4.19)	21.97 (5.00)	23.88 (6.43)
Ethnicity			
Aboriginal	3 (1.2)		1 (0.6)
Middle Eastern/Arab	6 (2.4)		3 (1.9)
Black	10 (4.0)		13 (8.0)
Chinese	56 (22.6)		28 (17.3)
European	59 (23.8)		85 (52.5)
Japanese	7 (2.8)		3 (1.9)
Korean	13 (5.2)		2 (1.2)
Latin American	11 (4.4)		12 (7.4)
South/South East Asian	34 (13.7)		9 (5.6)
White	115 (46.4)		N/A
Attachment Avoidance (*M*(*SD*), ɷ)	2.33 (0.82), .90	2.29 (0.84), .89	2.04 (0.57), .91
Attachment Anxiety (*M*(*SD*), ɷ)	2.95 (1.01), .90	2.73 (0.92), .90	2.84 (0.61), .91
Own Positive Emotions When Speaking (*M*(*SD*), ɷ)	5.84 (0.98), .87	5.90 (0.94), .87	5.31 (1.38), .94
Perceived Partner Positive Emotions When Speaking (*M*(*SD*), ɷ)	5.84 (1.03), .92	5.99 (0.98), .92	5.22 (1.42), .95
Own Positive Emotions When Listening (*M*(*SD*), ɷ)	6.10 (0.86), .87	6.11 (0.90), .87	5.38 (1.33), .94
Perceived Partner Positive Emotions When Listening (*M*(*SD*), ɷ)	5.99 (0.97), .92	6.09 (0.84), .92	5.26 (1.34), .95
Couple-level descriptives	Sample 1 (*N* = 124)	Sample 2 (*N* = 100)	Sample 3 (*N* = 79)
Relationship Length in months (*M*(*SD*))	24.66 (22.07)	18.63 (17.69)	31.08 (44.67)
Relationship Composition (*n*(%))			
Woman-man relationship	118.5^a^ (95.56)	90 (90)	71 (89.9)
Man-man relationship	0 (0)	2 (2)	1 (1.3)
Woman-woman relationship	4.5 (3.63)	6 (6)	4 (5.1)
Woman-gender diverse/not disclosed	1 (0.81)	1 (1)	1 (1.3)
Man-gender diverse/not disclosed	0 (0)	0 (0)	1 (1.3)
Gender diverse/not disclosed-gender diverse/not disclosed	0 (0)	1 (1)	1 (1.3)
Cohabitating (*n*(%))	37 (29.8)	25.5 (25.5)	38 (48.10)

*Note.* Individuals could select multiple ethnicities. We did not collect ethnicity information in Sample 2 (but this sample was recruited from the same population, using the same methods as Sample 1).

a One couple member did not report the same relationship configuration.

### Procedure

Study measures and procedures were approved by the University of Toronto (Samples 1 and 2) and Berkeley (Sample 3) Institutional Review/Research Ethics Boards. Couples attended an in-lab session where they first provided their informed consent to be videotaped and completed background questionnaires. Participants in Sample 3 were part of a larger study on couples’ communication (see Impett, 2019, for a list of related publications) and thus completed conversations in addition to those described below. Participants then engaged in two videotaped conversations with their partner in which we instructed one couple member—the speaker—to describe a time they “felt a lot of love for their partner and how they expressed it,” and the listener was able to react freely. Initial speaker/listener roles were randomized and couple members swapped speaker/listener roles for the second conversation. The length of conversations ranged from 15 s to 17 min 30 s (*M* = 3 min 2 s, *SD* = 1 min 59 s). Each member of the couple provided ratings of their own positive emotions and perceptions of their partners’ positive emotions following each conversation/each role.

### Measures

Participants completed additional questionnaires as part of larger studies, and we present below only measures included in this study. We present descriptive statistics along with *Omega* reliability estimates (calculated using the getOmega function in R; Gauvin et al., 2021) in Table 1.

#### Background Questionnaire

Participants indicated their demographic information (e.g., gender, age, and ethnicity) and relationship characteristics (e.g., relationship duration and cohabitation status).

#### Attachment

To assess attachment anxiety and attachment avoidance, participants in Samples 1 and 2 completed the Experiences in Close-Relationships–Revised questionnaire (ECR-R; Fraley, Waller, & Brennan, 2000), and participants in Sample 3 completed the Experiences in Close Relationships scale (ECR; Brennan et al., 1998). The difference in the choice of attachment measure across samples was an artifact of broader study goals and investigator preferences. The 5-point ECR and 7-point ECR-R scales assess agreement to 18 attachment-related anxiety statements (e.g., “I worry a lot about my relationships.”) and 18 attachment-related avoidance statements (e.g., “I get uncomfortable when a romantic partner wants to be very close.”) from *strongly disagree* to *strongly agree.* We calculated subscale scores by averaging all items, with higher scores indicating greater attachment anxiety and avoidance. We standardized scores prior to combining datasets to ensure the ECR and ECR-R values were comparable for analyses. Individuals’ attachment avoidance and attachment anxiety positively correlated at *r* = .32, *p* < .001, and couple members’ attachment scores were modestly correlated (anxiety: *r* = .15, *p* < .001, avoidance: *r* = .18, *p* < .001).

#### Self-Report and Partner-Perceived Emotions

This measure was adapted by Impett et al. (2010) for use in romantic relationships from a measure of social emotions (Srivastava et al., 2009). Participants rated on 7-point scales (1 = *not at all* to 7 = *a lot*) the extent to which they experienced six positive emotion triplets (happy/pleased/joyful; affectionate/loving/caring; proud/good about self; compassionate/sympathetic; grateful/appreciative; cared about/loved/connected) and six negative emotion triplets (e.g., anxious/nervous, contempt/disgust). We focus our analyses exclusively on positive emotions, given our hypotheses pertain to positive emotional processes. Furthermore, perceptions of partner’s negative emotions were very restricted in range (70%–85% of participants across samples reported floor values of 1); as such, we would not feel confident in the generalizability of any negative emotion effects and do not discuss negative emotions further (see Supplemental Materials section 1 for additional information).

### Analytic Approach

Because all three samples used identical methodology and measures, we combined them into one dataset, and controlled for sample in analyses (using effect coding). We analyzed the combined data using multilevel modeling (linear mixed model function in SPSS 29) guided by the Truth and Bias Model of Judgment (T. V. West & Kenny, 2011). While controlling for sample, we tested a two-level cross-classified model in which persons are nested within dyads crossed with conversation, to account for the fact that partners had the conversation with one another (Kenny et al., 2006). We estimated a random intercept and random slope for attachment avoidance^4^ for each couple, and treated dyads as indistinguishable (which allowed us to retain couples of all gender configurations), using a Compound Symmetry correlation metric to capture error variances (see OSF for syntax).

In our models, we predicted participants’ inference of their partners’ positive emotional state from (1) their partners’ actual positive emotions (i.e., truth), (2) their own positive emotions (i.e., assumed similarity), and the (3) lower and higher order interaction(s) of the truth and assumed similarity forces with (a) attachment avoidance, (b) conversation role (1 = speaker, −1 = listener), and (c) attachment anxiety. Before combining datasets, we centered the participants’ inference of their partners’ positive emotions, the truth, and assumed similarity around the grand mean of partners’ positive emotions in each sample (T. V. West & Kenny, 2011). Given this centering approach, the intercept in our models represents the directional bias (positive coefficient denoting over-perception, negative coefficient denoting under-perception), and the main effect of attachment avoidance represents whether higher avoidance is linked to over/underestimation of a partner’s emotions (hypothesis 1). The Avoidance × Truth interaction tests our key hypothesis (hypothesis 2) as to whether people higher in attachment avoidance are less empathically accurate, and the Avoidance × Assumed similarity interaction (exploratory hypothesis) explores whether avoidant people’s inferences about their partners’ positive emotions are swayed by their own positive emotions. To follow-up any significant interactions with attachment avoidance we conducted simple slopes analyses at one *SD* above and below the mean of avoidance (Aiken et al., 1991). For interactions involving conversation role we used dummy coding to examine the simple effects within each role (see S. G. West et al., 1996).

## Results

### Main Model

See Table 2 for all coefficients. There was a significant directional bias (i.e., the intercept), such that people tended to underestimate their partners’ positive emotions.^5^ Counter to hypothesis 1, this directional bias did not differ by whether individuals were high vs. low in attachment avoidance. Participants tended to accurately perceive their partners’ positive emotions (i.e., significant truth force), and projected their own positive emotions onto their partner, although as predicted these tendencies were qualified by attachment avoidance. We observed a significant random slope for attachment avoidance, signifying that the association between attachment avoidance and perceptions of a partner’s positive emotions differed by couple. The significant negative partial ICC value suggests that variance in perceptions of positive emotions was greater between individuals than between couples. Note, given our hypotheses pertained to the avoidance dimension of attachment, we limit our discussion of attachment anxiety to instances in which it influenced the key avoidance findings; however, interested readers can consult the OSF Supplemental Materials to view all anxiety results.

**Table 2.** Avoidance and Inferring a Partner’s Positive Emotions in Combined Dataset.

Parameter	*b*	*SE*	*df*	*p*	95% confidence interval	
					Lower bound	Upper bound
**Intercept**	**−0.04**	**0.02**	**295.91**	**.028**	**−0.07**	**0.00**
Sample effect code 1	0.06	0.02	263.32	.014	0.01	0.11
Sample effect code 2	**−**0.01	0.02	272.04	.590	**−**0.06	0.03
Anxiety	**−**0.06	0.02	795.99	.002	**−**0.09	**−**0.02
Avoidance	0.00	0.02	235.55	.920	**−**0.04	0.05
Truth	0.11	0.02	1,030.23	<.001	0.08	0.14
Assumed similarity	0.82	0.02	1,036.33	<.001	0.79	0.86
Conversation role	**−**0.01	0.01	314.13	.620	**−**0.03	0.02
Anxiety × Conversation role	**−**0.01	0.02	670.00	.721	**−**0.04	0.02
Avoidance × Conversation role	0.01	0.02	638.62	.599	**−**0.02	0.04
Truth × Conversation role	0.03	0.02	924.60	.050	0.00	0.06
Assumed similarity × Conversation role	**−**0.02	0.02	913.88	.177	**−**0.05	0.01
Anxiety × Truth	0.01	0.02	945.38	.510	**−**0.02	0.05
Avoidance × Truth	**−**0.05	0.02	551.89	.002	**−**0.09	**−**0.02
Anxiety × Avoidance	0.01	0.02	310.51	.593	**−**0.03	0.04
Anxiety × Assumed similarity	0.01	0.02	1075.17	.462	**−**0.02	0.05
Avoidance × Assumed similarity	**−**0.01	0.02	545.51	.624	**−**0.05	0.03
Avoidance × Truth × Conversation role	0.01	0.01	772.40	.655	**−**0.02	0.03
**Avoidance × Assumed** **similarity** × **Conversation role**	**−** **0.04**	**0.01**	**781.49**	**.004**	**−** **0.07**	**−** **0.01**
Anxiety × Truth × Conversation role	**−**0.02	0.02	763.88	.323	**−**0.04	0.01
Anxiety × Assumed similarity × Conversation role	0.02	0.02	864.25	.222	**−**0.01	0.05
**Avoidance** × **Anxiety** × **Truth**	**0.05**	**0.01**	**483.02**	**<.001**	**0.02**	**0.08**
Avoidance × Anxiety × Assumed similarity	**−**0.03	0.01	541.49	.047	**−**0.06	0.00
Avoidance × Anxiety × Conversation role	0.01	0.01	625.88	.670	**−**0.02	0.03
Avoidance × Anxiety × Truth × Conversation role	**−**0.02	0.01	699.51	.127	**−**0.04	0.01
Avoidance × Anxiety × Assumed similarity × Conversation role	0.00	0.01	761.71	.731	**−**0.02	0.03

*Note.* Conversation role = speaker/listener role (1 = speaker, −1 = listener). In sample effect code 1, Sample 1 = 1, Sample 2 = 0, Sample 3 = −1. In sample effect code 2, Sample 1 = 0, Sample 2 = 1, Sample 3 = −1. There was a statistically significant random effect of Avoidance (*b* = 0.03, *SE* = .01, *p* < .001 95% CI [0.02, 0.06]), and variance in perceptions of positive emotions was greater within- versus between-dyads (CSR: *b* = −.17, *SE* = 0.05, *p* = .002, 95% CI [−.27, −.06]). Key effects are bolded. The 3-way Avoidance × Anxiety × Assumed similarity interaction was statistically significant; however, it is not bolded as the follow-up 2-way Avoidance × Assumed similarity interaction was not statistically significant at either high (+1*SD*) or low (−1*SD*) Anxiety, and as such is not discussed further (interested readers can see Supplemental Materials 4).

#### Avoidance and Accuracy

**Figure 1.**
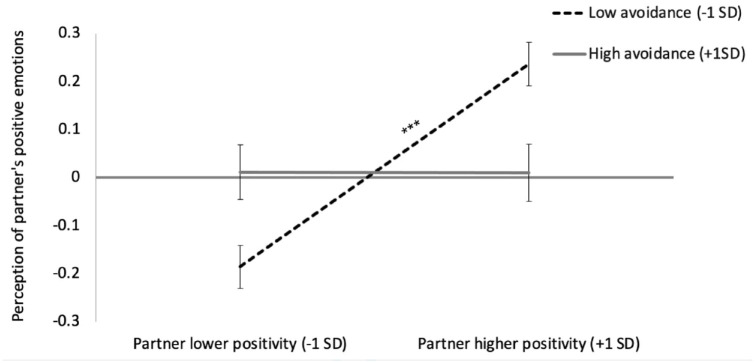
Attachment Avoidance and Accuracy in Inferring a Partner’s Positive Emotions at Low (−1*SD) Levels of Attachment Anxiety.* *Note.* ***p < .001. The error bars represent standard errors. The value of 0 represents no directional/mean-level bias. Partner lower in positivity (−1*SD*) corresponds to a positive emotion score of 4.25 to 4.84 (there is a range given “truth” was centered by sample), whereas partner higher in positivity (+1*SD*) corresponds to a score of 6.36 to 6.95. The significant positive slope for those low in avoidance indicates accuracy (i.e., correctly differentiating low from high partner positivity) whereas the flat, nonsignificant slope for high avoidance suggests inaccuracy.

Although participants tended to accurately perceive their partners’ positive emotions, there was an effect of avoidance on accuracy which was further moderated by anxiety, whereby avoidance moderated accuracy for those low (*b* = −.10, *SE* = .02, *p* < .001) but not high (*b* = −.003, *SE* = .02, *p* = .90) in anxiety. In Figure 1, we depict how avoidance significantly moderated accuracy for those low in anxiety, regardless of conversation role. As depicted by the relatively flat slope, those high in avoidance were not accurate (*b* = .01, *SE* = .04, *p* = .80) in inferring their partners’ positive emotions, but, as depicted by the positive slope, those low in avoidance were accurate (*b* = .20, *SE* = .03, *p* < .001; see Supplemental Materials section 3 for full models). In other words, at low levels of attachment anxiety, for those high in avoidance, their partner’s true positive emotions were not significantly associated with their perceptions of their partner’s emotions (whereas for those low in avoidance their perceptions were significantly related to their partner’s true emotions).

#### Avoidance and Assumed Similarity

We observed a three-way interaction in which assumed similarity was qualified by avoidance and conversation role, such that there was an Avoidance × Assumed similarity interaction when participants were in the speaker role (*b* = −.05, *SE* = .02, *p* = .025) but not the listener role (*b* = .03, *SE* = .02, *p* = .17; see Supplemental Materials section 4 for full models). When in the speaker role, both those high and low in avoidance projected their positive emotions onto their partner, although those high in avoidance projected less (*b* = .75, *SE* = .03, *p* < .001) than those low in avoidance (*b* = .85, *SE* = .03, *p* < .001). We also observed a just significant (*b* = −.03, *SE* = .01, *p* = .047) three-way interaction of Avoidance × Anxiety × Assumed similarity; however, neither of the two-way Avoidance × Assumed similarity follow-ups were significant (*p*s = .06−.46). As such, anxiety does not meaningfully influence the effect of avoidance on the tendency to assume similarity.

#### Interim Summary

Overall, there was a tendency for people to accurately infer their partners’ positive emotions (i.e., truth) and base their inferences on their own emotions (i.e., assumed similarity). Yet, as predicted, these effects differed by attachment avoidance levels as summarized in Table 3.

**Table 3.** Summary of Attachment Avoidance as a Moderator When Inferring A Partner’s Positive Emotions.

Force	Moderation summary	Hypothesis
Directional bias	The tendency to underestimate did not differ by avoidance.	Failed to support hypothesis 1
Truth	High avoidant individuals were less accurate in inferring their partners’ positive emotions relative to low avoidant individuals (when listening or speaking) This effect was qualified by attachment anxiety such that it was not observed at high levels of anxiety (+1*SD*).	Support for hypothesis 2
Assumed similarity	High avoidant individuals projected less of their positive emotions when they were sharing their own time of love (speaking), relative to low avoidant individuals.	Exploratory hypothesis: Partial support for higher avoidance and lower projection

### Alternative Explanations

We conducted additional analyses to ensure the effects presented in Table 3 were not attributable to other factors associated with empathic accuracy such as the judger’s age (Ruffman et al., 2008), relationship length (Thomas et al., 1997), relationship satisfaction (Sened et al., 2017), or commitment (Kilpatrick et al., 2002). Our pattern of results remained robust to these variables (for analyses and results, see Supplemental Materials section 5a–d). Moreover, whether it was the couple’s first or second conversation did not alter the effects (see Supplemental Materials section 5a). We also explored gender as a moderator of the effects in Table 3 (see Supplemental Materials section 5f), and gender did not interact with avoidance to predict directional bias (*b* = .01, *SE* = .02, *p* = .537) in the overall model, nor did gender interact with avoidance to predict assumed similarity in the speaker role (*b* = .01, *SE* = .02, *p* = .812). Regarding the effects for accuracy, there was a significant Avoidance × Truth interaction for both men and women. For transparency, there was a 5-way interaction between Avoidance × Anxiety × Truth × Speaker/Listener Condition × Gender, whereby the pattern of results mentioned in Table 3—higher avoidance is associated with poorer accuracy than lower avoidance—was observed in seven of the eight follow-up analyses. The one exception was that, for women in the speaker condition who were low on anxiety, higher avoidance was associated with greater accuracy than lower avoidance. Given this is one anomaly among a robust pattern of results, we hesitate to make too much of this gender difference.

### Replication of Effects Across Samples

To add greater confidence to our findings, we then tested whether our aforementioned effects (summarized in Table 3) replicated in each individual dataset. Consistent with recommendations for analyzing pooled data, we conducted a fixed-effects Integrative Data Analysis (Curran & Hussong, 2009; Hussong et al., 2013), in which we re-ran our analyses moderating each term by sample (using two effect-coded variables to represent the three samples; see S. G. West et al., 1996). This allowed us to determine whether our presented effects were consistent across the three samples. Because sample is represented by two fixed-effects coded variables in the model, we refer to ps below to encompass both of these codes. When there was a significant moderation by sample, we conducted follow-up analyses within each sample using dummy coding (see Supplemental Materials section 6a–d for all models).

Avoidance did not interact with sample to predict the directional bias (see sheet 6A in supplemental *p*s >.073). Although the Truth × Avoidance × Anxiety interaction was moderated by sample, follow-ups were nonsignificant across each of the three samples (*p*s = .86, .11, and .17, respectively). The pattern of the Avoidance × Truth interaction, however, replicated across two out of the three samples, such that those high in avoidance were not accurate in perceiving their partners’ positive emotions (Sample 2: *b* = −.01, *SE* = .06, *p* = .840; Sample 3: *b* = .01, *SE* = .04, *p* = .882) whereas those low in avoidance were accurate (Sample 2: *b* = .24, *SE* = .05, *p* < .001; Sample 3: *b* = .19, *SE* = .04, *p* <.001). However, in Sample 1 the Avoidance × Truth interaction was nonsignificant. Finally, the three-way interaction between assumed similarity, avoidance, and conversation role did not differ across the three samples (*p*s > .28).

### Overall Summary

Taken together, our results suggest that the tendency for avoidant individuals to be less accurate at reading a romantic partner’s positive emotion is fairly robust across covariates and samples, as is avoidant individuals’ tendency to project less of their positive emotions when speaking to their partner (see Table 4).

**Table 4.** Summary of Robustness of Results Across Alternative Explanations.

Force	Result	Moderations with attachment anxiety	Control variables	Replication
Truth	High avoidant individuals were less accurate in inferring their partners’ positive emotions relative to low avoidant individuals (when listening or speaking).	This effect was qualified by attachment anxiety such that it was not observed at high levels of anxiety (+1*SD*).	Results hold across 6 covariates with some additional qualifications for gender	Replication in 2/3 samples
Assumed similarity (exploratory)	High avoidant individuals projected less of their positive emotions when they were sharing their own time of love (speaking), relative to low avoidant individuals.	Not moderated by anxiety	Results hold across 6 covariates	Replication in 3/3 samples

## Discussion

Our study sought to extend work on attachment and empathic accuracy to a positive context. Combining three samples, we investigated how attachment avoidance affected individuals’ accuracy in perceiving their partner’s positive emotions after the couple shared feelings of love. Overall, couple members tended to accurately perceive their partner’s positive emotions, although they tended to underestimate them (consistent with a large body of similar findings in relationship science; see Fletcher & Kerr, 2010; LaBuda & Gere, 2023). They also tended to project their own emotions onto their judgments of their partner’s emotions, consistent with much work in relationship science (e.g., Clark et al., 2017; see LaBuda & Gere, 2023). Yet, as expected, these patterns differed by individuals’ attachment avoidance. Consistent with our hypotheses, individuals higher in avoidance showed lower empathic accuracy in that they did not pick up on their partner’s positive emotions to the same degree as secure individuals—regardless of whether they were sharing, or listening to their partner share, a time of love. This pattern of results was limited to individuals low in attachment anxiety, perhaps suggesting that in this highly positive intimate context, concerns about a partner’s regard (anxiety) overwhelmed concerns about independence (avoidance). Counter to our predictions, however, avoidant individuals underestimated their partners’ positive emotions to a similar degree as more secure individuals. We also observed that when avoidant individuals shared a time of love, they were less likely to ground their judgments of their partner’s emotions in their own emotions (i.e., assume similarity) than those lower in avoidance. When listening to their partner share love, the tendency to assume similarity did not differ based on avoidance.

Our findings echo meta-analytic effects when judging positive interactions, in that our observed overall assumed similarity/projection effect (*r* = .83) was stronger than our accuracy effect (LaBuda & Gere, 2023). Our effect size for accuracy at high avoidance is *r* = .12, whereas the effect size for accuracy at low avoidance is *r* = .22, which is remarkably similar to the average effect size β = .16 reported by LaBuda and Gere (2023). Like LaBuda and Gere (2023), we did not find that gender played a reliable role in accuracy levels.

Of note and as depicted in Figure 1, individuals high in avoidance showed lower accuracy both when their partners were truly lower or higher in positive feelings. That is, it was not simply the case that avoidant individuals exhibited lower accuracy when their partners showed strong intimacy cues. This failure to differentiate between less and more intimacy might suggest that avoidant individuals are not paying attention to their partner’s positive feelings. Our results are consistent with other findings regarding avoidance and positive stimuli, such as that avoidant individuals do not differentiate their feelings of connection between a warm- versus cold-behaving confederate (Philipp-Muller & MacDonald, 2017).

Our work cannot directly inform whether avoidant individuals are insensitive to positive cues or purposely disregard such cues. Nevertheless, we believe the latter interpretation may be more likely because, as depicted in Figure 1, more avoidant individuals had higher perceptions of positivity than secure individuals when their partner was truly lower in positivity (keeping in mind “lower” positivity is still above the scale midpoint). That is, when a partner does not show strong cues of love, there is less of a need for avoidant individuals to guard against this intimacy, and thus they could acknowledge their partner’s positivity. Perhaps these moderate cues of positivity are a “comfort zone” for highly avoidant individuals. Although we suspect sharing a time of love is more threatening to a person high in avoidance than receiving a partner’s love, their lower accuracy in both conversations may reflect both contexts’ objectively highly intimate nature. Likewise, observing that avoidant individuals project their own emotions less than secures when sharing a time of love further supports that avoidant individuals’ perceptions of positive cues may be defensively motivated. That is, given those high in avoidance self-report they are uncomfortable with intimacy and are less likely to express emotions (e.g., Brandão et al., 2020; Kerr et al., 2003), disclosing a time of love (relative to receiving feelings of love) is a particularly vulnerable state in which they may especially need to disengage from a partner via lower projection. This projection effect is inconsistent with past work with negative emotions that found null projection effects for avoidant individuals (Overall et al., 2015; Sadikaj et al., 2018). One interpretation is that avoidant individuals may project their own feelings in contexts that benefit their desire for distance. Of course, future work is needed to disentangle these possibilities more conclusively.

### Implications

Our results contribute to both attachment theory and the Empathic Accuracy Model (Ickes & Simpson, 1997, 2001). We identify intimacy-related contexts as empathic accuracy “danger zones” for avoidant individuals, adding to the previously identified “danger zones” of jealousy and conflict contexts (Simpson et al., 2011). Our findings that avoidant individuals are not accurate in this positive context echo findings using diverse methods (e.g., memory studies; Gentzler & Kerns, 2006; brain imaging Vrtička et al., 2008) that illustrate lower responsiveness to positive stimuli, and our relatively large sample size enhances confidence in our findings.

Unlike past work in negative contexts (Simpson et al., 2011), we observed lower accuracy for people high in avoidance, particularly when they were low (but not high) in attachment anxiety. Many studies do not assess how avoidance and anxiety combine to influence key relational outcomes, or are under-powered to detect effects (cf. Park et al., 2019). Thus, our study adds important context to broader work on addressing avoidant attachment defenses (Park et al., 2019), by reiterating the necessity of differentiating the patterns of “dismissive” (i.e., low anxiety) and “fearful” (i.e., high anxiety) avoidants. For instance, interventions to increase highly avoidant individuals’ empathic accuracy may not be beneficial for those who are simultaneously high in attachment anxiety.

It is unfortunate that those high in avoidance failed to pick up on their partner’s positive emotions during this intimate context. Avoidant people tend to feel less connected to their partners (see meta-analysis by Li & Chan, 2012). But, positive relationship factors such as perceiving gratitude or appreciation from a romantic partner (Park et al., 2019; Schrage et al., 2022), intimacy-related experiences (Stanton et al., 2017), and strong cues of support (Girme et al., 2015) particularly benefit avoidant individuals—so long as avoidant individuals’ feelings of autonomy can be protected (e.g., Girme et al., 2019; Simpson & Overall, 2014). In other words, correctly picking up on their partner’s feelings of love could bolster avoidant individuals’ connections to their partner.

### Strengths, Limitations, and Future Directions

Previous research examining the role of attachment avoidance in shaping empathic accuracy has exclusively focused on negative contexts (e.g., relationship threat; Ickes & Simpson, 1997, 2001; relationship conflict Overall et al., 2015). The current study is the first to examine the moderating role of attachment avoidance on empathic accuracy during conversations about love. For individuals high in attachment avoidance, positive relationship contexts and signs of intimacy may be their true “danger zone” during which they deactivate their attachment systems (Mikulincer & Shaver, 2007). As such, conversations that are rated as more intimate—such as love conversations—should theoretically produce lower accuracy among highly avoidant individuals. Examining the content of couples’ love conversations allowed us to further test our hypothesis that a difference in motivation might drive avoidant individuals’ lower accuracy. However, our studies only examined empathic accuracy in a conversation about love and did not test whether lower accuracy extended to other more intimate relationship contexts (e.g., conversations about shared positive experiences, conversations about a time of excitement). Future research should examine whether our findings generalize to other intimate relationship contexts. Furthermore, to more stringently test our hypothesis that lower accuracy is a motivated defense, future research could examine empathic accuracy of avoidant individuals in a positive conversation that was not relationship relevant; for example, if members of the couples described their favorite hobby, or a positive aspect about their day. In nonrelationship relevant positive conversations, the conversations should not be as intimate, and as such avoidant individuals should feel the need to defend themselves and therefore should not show such low accuracy.

The current study combined data from three separate samples, which resulted in a final combined sample that was varied (community sample, student sample, different geographic locations and ethnicities, individuals in same and mixed-gender relationships, etc.). Unfortunately, we did not have a sufficient sample size to examine effects across demographic groups (e.g., race/ethnicity and relationship type), and the results may not generalize to other relationship constellations, such as those in consensual nonmonogamous relationships (Sakaluk et al., 2021). The moderating effect of attachment avoidance was, however, robust to covariates of age, gender (for the most part), conversation order, relationship commitment, satisfaction, and length. Yet, it is also possible other contextual variables we did not assess could influence empathic accuracy, and account for the nonreplication of the Avoidance × Truth in Sample 1. For instance, those high in prosocial orientation (Côté et al., 2011) and lower in socioeconomic status (Kraus et al., 2010) tend to be more accurate, and many other facets of the target and perceiver influence accuracy (see review by Hodges et al., 2015). Moreover, it is possible findings for “fearful” individuals could change to resemble “dismissive” findings if the conversations became habitual, allowing the partner’s affection to become more autonomy threatening. Although our pattern of results held after controlling for relationship length, the average relationship length was fairly short (less than 3 years), and the observed results may not generalize to couples in longer-term relationships. We would anticipate that longer-term couples might be less accurate than the current participants overall (Kilpatrick et al., 2002); however, the moderating effect of attachment avoidance should persist.

Our study did not examine the long-term or dyadic consequences of lower empathic accuracy in relationships. Individuals who are higher in attachment avoidance and their partners have lower relationship quality across 1 year (Tan et al., 2012), and increased risk of relationship dissolution (Le et al., 2010). A longitudinal study design would be valuable to determine whether the lower empathic accuracy of avoidant individuals contributes to subsequent relational difficulties, such as increased conflicts, lower commitment, or a greater chance of relationship dissolution. Such a study could be enhanced by looking at whether couple members’ similarity in empathic accuracy has repercussions for their satisfaction, and if a secure partner’s accuracy can compensate for the lower accuracy of an avoidant individual (i.e., a strong link effect; Sasaki et al., 2023). Focusing on the interplay between both partners’ accuracy is an exciting area for future research. Future research could also examine ways for partners to help avoidant individuals feel more comfortable during love conversations, such as partners facilitating autonomy (Girme et al., 2019). Focusing on the partners’ behaviors during love conversations would also help inform whether the partner is in any way contributing to the lower accuracy of avoidant individuals (e.g., Noller & Ruzzene, 1991).

Another major strength of the current study was having couples reflect on conversations that had just occurred in the laboratory, minimizing the recall window for retrospective reporting. However, we did not assess the accuracy of a partner’s moment-to-moment thoughts and feelings during the conversation (e.g., Ickes, 1997), which might have reduced the influence of individual differences like attachment avoidance, which is an exciting possibility for future work. Conversations in laboratory environments tend to be rated as highly similar to those outside laboratory environments (e.g., Gauvin et al., 2019; Gottman, 1979), thus, we would expect that accuracy should be fairly representative as partners are unlikely to have been “thrown off” by atypical communication patterns. However, as avoidant people may not spontaneously declare love, it is also possible that the conversation reflected a more contrived scenario for avoidant individuals. Moreover, couples who sign up to participate in dyadic studies may be particularly satisfied (Barton et al., 2020; Park et al., 2021), and somewhat comfortable discussing their romantic relationship, meaning effects observed from the avoidant individuals who agreed to participate may not generalize to all avoidant individuals. Future research could examine whether avoidant and nonavoidant individuals differ in how representative they rate in-lab conversations compared to their typical at-home communications.

## Conclusion

Sharing feelings of love is an opportunity for couples to grow closer. However, by being less attuned to their partner’s cues of positivity, avoidant individuals may be missing out on the connection that could ultimately help them have more satisfying relationships.

## Notes

1. Note, given the specifications of the Truth and Bias Model of Judgment, a lack of accuracy is represented by a nonsignificant “truth” effect, rather than a negative coefficient (T. V. West & Kenny, 2011).

2. See Supplemental Materials for additional manipulation/condition information.

3. Assuming the default values for the correlation between actor and partner variables, correlated errors (*r* = .3), and an alpha level of .05.

4. Given the complexity of the model, the model would not converge with each predictor entered as a random slope. We prioritized estimating the random effect of avoidance given its centrality to our predictions.

5. However, we encourage readers to interpret the overall directional bias with caution as these effects were not robust across samples. Interested readers can see Supplemental Materials section 6a–d for the directional bias within each sample.
